# Screening and Structure–Activity Relationship of D2AAK1 Derivatives for Potential Application in the Treatment of Neurodegenerative Diseases

**DOI:** 10.3390/molecules27072239

**Published:** 2022-03-30

**Authors:** Oliwia Koszła, Przemysław Sołek, Piotr Stępnicki, Agnieszka A. Kaczor

**Affiliations:** 1 Department of Synthesis and Chemical Technology of Pharmaceutical Substances with Computer Modeling Laboratory, Faculty of Pharmacy, Medical University of Lublin, 4 A Chodzki St., 20-093 Lublin, Poland; piotrstepnicki93@gmail.com; 2 Department of Biotechnology, Institute of Biology and Biotechnology, University of Rzeszow, 1 Pigonia St., 35-310 Rzeszow, Poland; pp.solek@gmail.com; 3 School of Pharmacy, University of Eastern Finland, Yliopistonranta 1, P.O. Box 1627, FI-70211 Kuopio, Finland

**Keywords:** D2AAK1 derivatives, neurodegeneration, proliferation, redox balance, screening

## Abstract

Neurodegenerative and mental diseases are serious medical, economic and social problems. Neurodegeneration is referred to as a pathological condition associated with damage to nerve cells leading to their death. Treatment of neurodegenerative diseases is at present symptomatic only, and novel drugs are urgently needed which would be able to stop disease progression. We performed screening of reactive oxygen species, reactive nitrogen species, glutathione and level intracellular Ca^2+^. The studies were assessed using one-way ANOVA of variance with Dunnett’s post hoc test. Previously, we reported D2AAK1 as a promising compound for the treatment of neurodegenerative and mental disorders. Here, we show a screening of D2AAK1 derivatives aimed at the selection of the compound with the most favorable pharmacological profile. Selected compounds cause an increase in the proliferation of a hippocampal neuron-like cell line, changes in the levels of reactive oxygen and nitrogen forms, reduced glutathione and a reduced intracellular calcium pool. Upon analyzing the structure–activity relationship, we selected the compound with the most favorable profile for a neuroprotective activity for potential application in the treatment of neurodegenerative diseases.

## 1. Introduction

Neurodegenerative and mental diseases affect millions of people worldwide. They are one of the most common causes of human inability to function in everyday life, with a direct impact at the professional or social level of patients [1]. Neurodegeneration is progressive and is characterized by the loss of neurons and impaired motor and cognitive functions. Consequently, the functional and structural damage to neurons may result in cell death [2]. To date, there are no effective therapeutic methods that would eliminate or alleviate the factors causing the progression of neurodegenerative diseases [3]. For this reason, the development of effective therapies constitutes the global neuroscience research challenge [1]. The pharmaceuticals used so far are characterized by moderate effectiveness; therefore, the development and synthesis of new compounds with potential neuroprotective properties is urgently needed [4,5]. Current screening methodologies in novel drug discovery are performed to select compounds with the most desirable properties. Common high throughput screening assays provide data about potential cytotoxicity, genotoxicity and antiproliferative properties [6]. In turn, our previous studies showed that the D2AAK1 compound displays antipsychotic, anxiolytic and procognitive effects in respective animal models. This compound has been identified in structure-based virtual screening as a multi-target ligand of aminergic G protein-coupled receptors (GPCRs) [7]. Moreover, the compound is characterized by neuroprotective properties as well as stimulation of neuron growth and survival [8,9]. This important finding suggests its potential application in the treatment of neurodegenerative and mental disorders.

**Figure 1:**
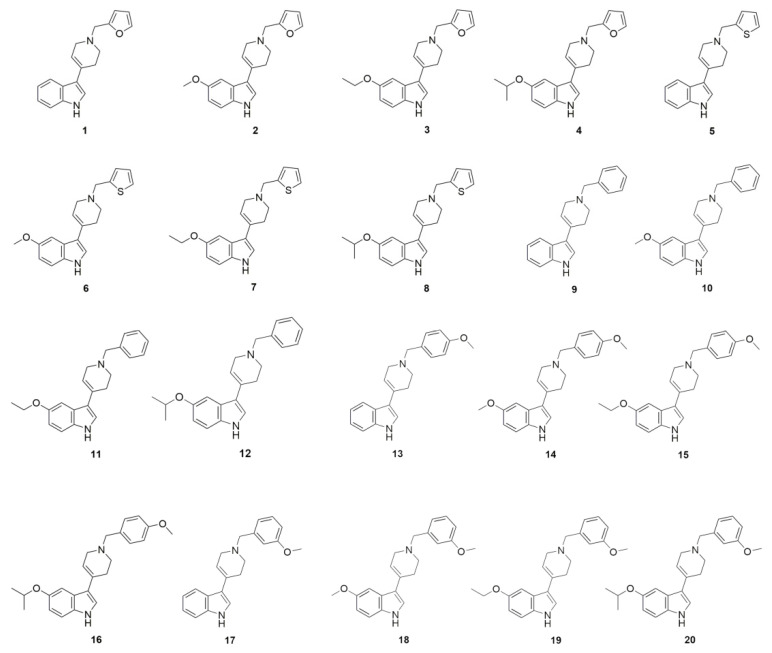
2 D structures of D2AAK1 derivatives.

Here, we screened D2AAK1 derivatives **1**–**20** (Figure 1) [10] for the selection of the most optimal pharmacological profile of compounds as new pharmaceutical substances for the treatment of neurodegenerative diseases. We observed that the compounds cause the increase in hippocampal neurons’ proliferation rate, changes in the level of reactive oxygen species (ROS), reactive nitrogen species (RNS), or the non-enzymatic cell protection mechanism (reduced glutathione—GSH) and depleted intracellular calcium pool. Upon analyzing the structure–activity relationship of D2AAK1 and its derivatives, it may be concluded which compounds from among all implemented structural features are the most favorable for neuroprotective activity.

## 2. Results and Discussion

The main cause of neurodegenerative diseases is the death of nerve cells. This phenomenon is mainly initiated by oxidative and nitrosative stress. The consequences of a redox imbalance can be highly destructive and include DNA damage and protein or lipid degradation, which inevitably leads to cell death. Moreover, ROS and RNS can activate interacting mechanisms including disturbance of intracellular calcium levels (Ca^2+^) [11]. Therefore, the screening of compounds **1**–**20** (Figure 1) included such assays as cell viability, levels of reactive oxygen and nitrogen species, reduced glutathione and reduced intracellular calcium.

Previously, we provided evidence of the unique properties of the lead structure D2AAK1 in terms of neuronal growth stimulation and cell survival promotion. The events observed were related to the up-regulation of neurotrophic factors, CAMKI kinase and mechanisms related to cell proliferation confirmed by BrdU incorporation into newly synthesized DNA [8]. Moreover, D2AAK1 showed antioxidant properties, decreased the levels of ROS and RNS and prevented excitotoxicity by decreasing intracellular calcium and NMDA levels. The lead structure shows neuroprotective properties thanks to protection of nerve cells against DNA damage and high temperature, not disturbing the cell cycle profile at the same time [9].

**Figure 2:**
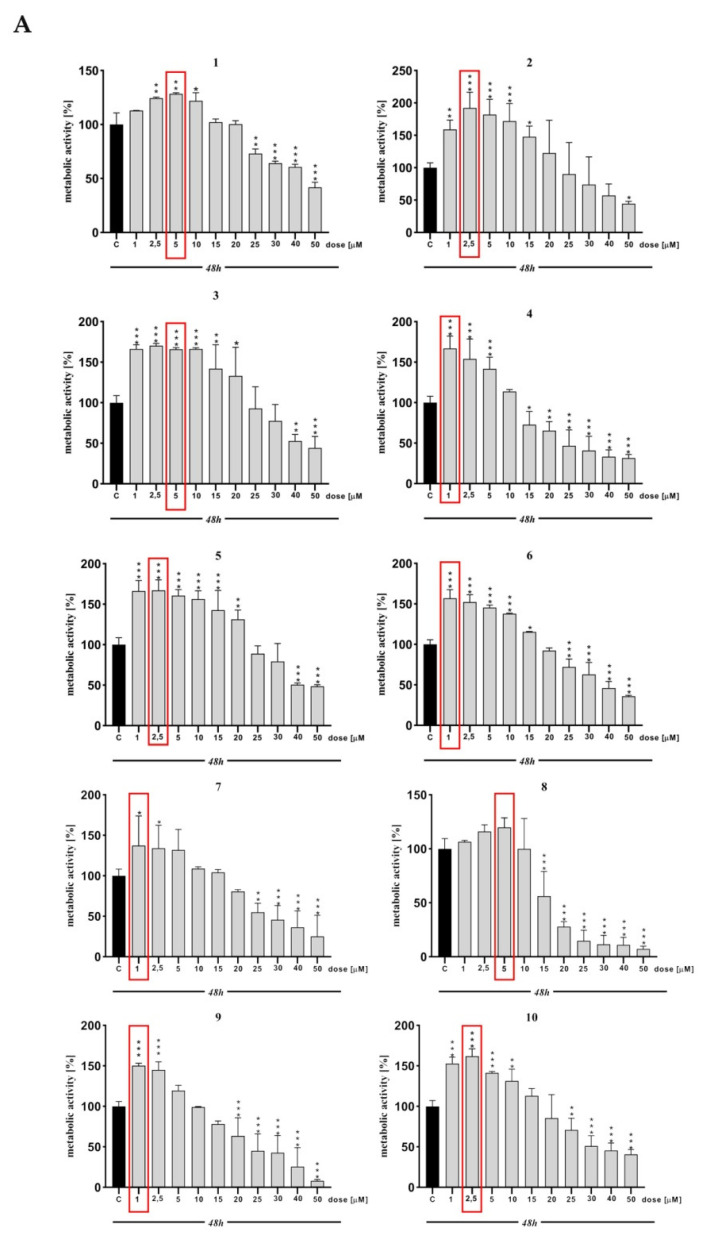

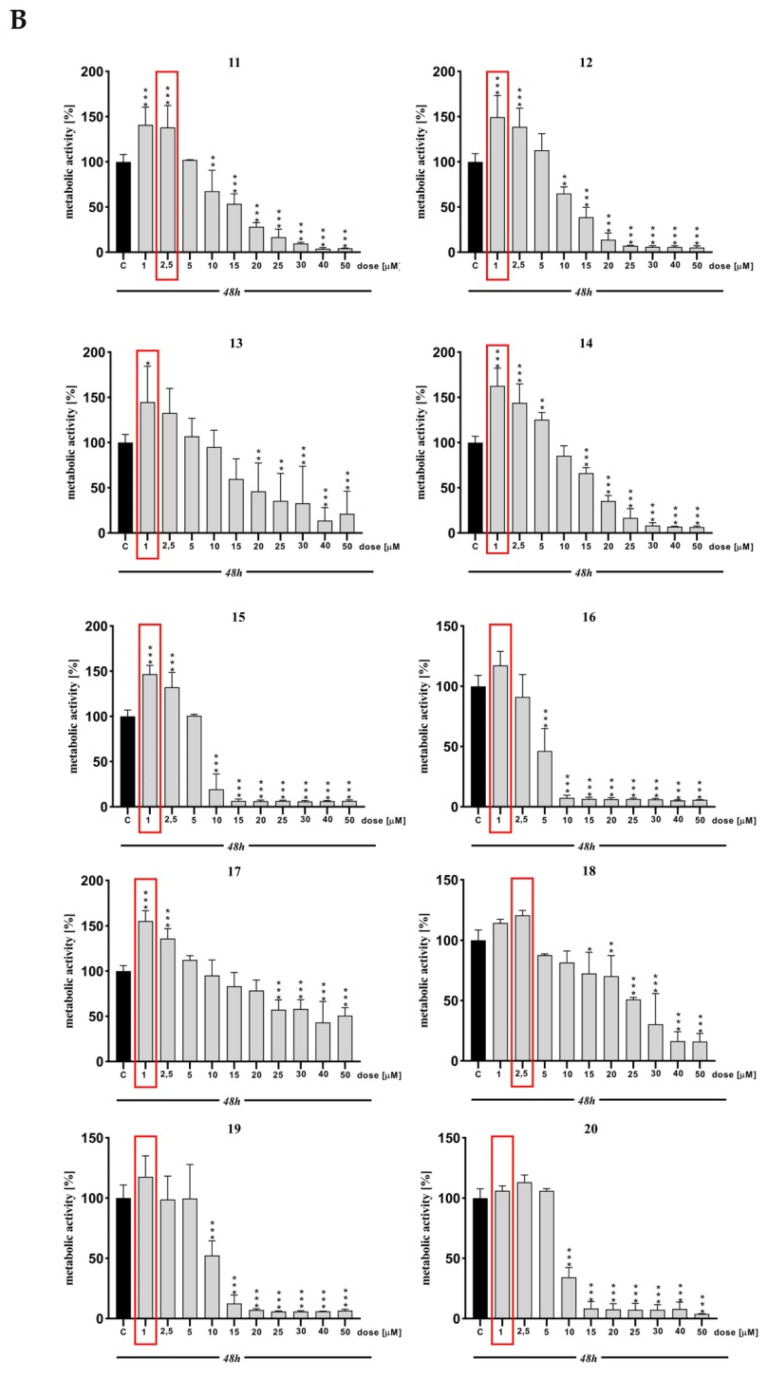
(**A**). Metabolic activity of HT-22 cells treated with compounds **1**–**10** for 48 h. All experimental groups were compared using one-way ANOVA of variance with Dunnett’s post hoc test; mean ± SD, *n* = 9. Statistically significant results are displayed as: * *p* < 0.05; ** *p* < 0.01; *** *p* < 0.001. (**B**) Metabolic activity of HT-22 cells treated with compounds **11**–**20** for 48 h. All experimental groups were compared using one-way ANOVA of variance with Dunnett’s post hoc test; mean ± SD, *n* = 9. Statistically significant results are displayed as: * *p* < 0.05; ** *p* < 0.01; *** *p* < 0.001.

In the case of screening related to cell viability and their proliferation in all sets tested, we observed a dose-dependent decrease in the cell metabolic activity. In detail, the highest proliferation rate was observed in the compounds possessing furanylmethyl substituents linked to the tetrahydropyridine moiety (Figure 2A, compounds **1**–**4**). Among this group and in general, in all sets tested, the highest metabolic activity occurred in the derivative with a methoxy substituent at the C5 position (Figure 2A, compound **2**) of the indole (***, *p* < 0.001). This activity lowered with the increasing size of alkoxy groups (Figure 2A, compounds **3**–**4**), although the lowest activity was interestingly shown by the compound with no substituent in this position (Figure 2A, compound **1**) (**, *p* < 0.01). Contrary to thiophenylmethyl moiety, the compound with an unsubstituted indole (Figure 2, compound **5**) exhibited the highest proliferation rate (***, *p* < 0.001). The alkoxy group installed (Figure 2A, compounds **6**–**8**) led to decreasing metabolic activity with a similar tendency to furanylmethyl fragment, namely, the bigger the substituent, the lower the activity. Among the derivatives with benzyl groups (Figure 2A,B, compounds **9**–**12**) at the tetrahydropyridine fragment, again those with small alkoxy substituents (Figure 2A, compound **10**) (***, *p* < 0.001) or with no substituent (Figure 2A, compound **9**) (***, *p* < 0.001) at the C5 position of the indole showed the highest proliferation while implementing bigger (ethoxy or isopropoxy) groups (Figure 2B, compounds **11**–**12**) caused decreases in cell metabolic activity. Applying the 4-methoxy group to the benzyl substituent (Figure 2B, compounds **13**–**16**) does not significantly change the tendencies in affecting metabolic activity. Derivatives with a 3-methoxybenzyl substituent (Figure 2B, compounds **17**–**20**) showed the lowest proliferation rate among other substitution options at the tetrahydropyridine moiety. Nonetheless, the trend remained the same—with the increasing bulkiness of the alkoxy substituent at the C5 indole (Figure 2B, compounds **19**–**20**), the metabolic activity decreased.

The most favorable for enhancing the proliferation seems to be the compounds with methoxy substituents at the C5 position of the indole (Figure 2A,B, compounds **2**, **6**, **10**, **14** and **18**). With the increasing size of this moiety, the cell metabolic activity decreased. Considering different chemical groups attached to the tetrahydropyridine fragment, smaller, 5-membered ring substituents (Figure 2A, compounds **1**–**8**) are more beneficial than 6-membered rings (Figure 2A,B, compounds **9**–**20**). As it was already emphasized, ethoxy and isopropoxy groups (Figure 2A,B, compounds **3**–**4**, **7**–**8**, **11**–**12**, **15**–**16** and **19**–**20**) are unlikely to enhance proliferation rates. Furthermore, compounds with these substituents exhibit toxicity at higher concentrations, with a more pronounced effect within derivatives possessing bigger, 6-membered rings (Figure 2A,B, compounds **9**–**20**) at the tetrahydropyridine, rather than 5-membered ring groups (Figure 2A, compounds **1**–**8**).

Based on the MTT assay, single concentrations of compounds that increase the HT-22 cells metabolic activity were selected for all further studies (marked with a red frame) (Figure 2A,B).

The available literature’s data indicate that antipsychotic drugs improve the survival of nerve cells and support their division [12,13]. Increasing the proliferation of neurons and creating new neural connections is a key point in the treatment of neurodegenerative and mental diseases [2]. Upon analyzing the structure–proliferation relationship of the tested compounds, it can be clearly stated that the lead structure of D2AAK1 causes the highest increase in proliferation [8] compared to its derivatives. The increase in proliferation can be induced as a result of the activation of mechanisms related to the hormesis effect. Hormesis describes dose-response relationships often characterized by stimulation of proliferation by low dose [14]. However, the cell is characterized by the presence of various protective mechanisms. In fact, their action determines cell survival or death. One of the cell death pathway indicators is an increase in the intracellular calcium pool [15], but the function of calcium is not limited to cell death only. Calcium release plays an important role in the control of neurite growth, synaptic plasticity, secretion and neurodegeneration [16]. Additionally, for neurons, levels of calcium are crucial due to its participation in the transmission of depolarizing signals and synaptic activity [17].

**Figure 3:**
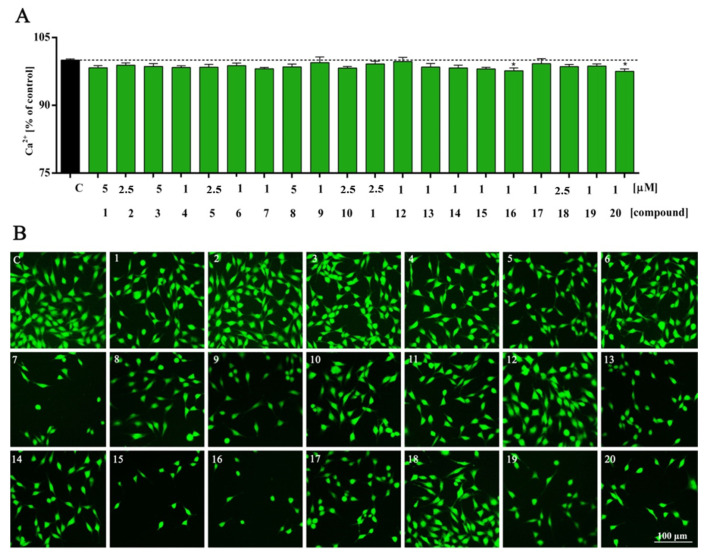
Intracellular calcium (Ca^2+^) level of HT-22 cells treated with compounds **1**–**20** (**A**) for 48 h and representative images (**B**). All experimental groups were compared using one-way ANOVA of variance with Dunnett’s post hoc test; mean ± SD, *n* = 9. Statistically significant results are displayed as: * *p* < 0.05. Green fluorescence—FITC. Magnification of the objective lens 10×.

Here tested compounds did not alter the levels of intracellular calcium. The only derivatives that cause statistically significant changes are the ones with isopropyl groups at the C5 indole and 3-methoxy or 4-methoxybenzyl substituents at the tetrahydropyridine fragment (Figure 3, compounds **16**, *p* = 0.0486; **20**, *p* = 0.0295). In other cases, the results were at the control level or slightly below; however, they were not statistically significant (Figure 3).

The interaction between calcium level and nitric oxide (NO) in nerve cells has many physiological as well as pathophysiological aspects. Nitric oxide is also considered to be an important effector of calcium accumulation through the potential-dependent Ca^2+^ mitochondrial channel. Overall, mitochondria are central in the pathogenesis of neurodegenerative diseases due to ROS overproduction or specifically impaired Ca^2+^-buffering capacity [18,19]. In turn, excessive increase in cytosolic Ca^2+^ concentration can result in redox imbalance and impaired mitochondrial function and cellular bioenergetics. Most importantly, calcium level dysregulation or mitochondrial impairment may also contribute to aberrant protein folding [17,20]. Thus, in neurodegenerative processes, the ability of neurons to maintain adequate energy levels or redox balance is an extremely important aspect.

**Figure 4:**
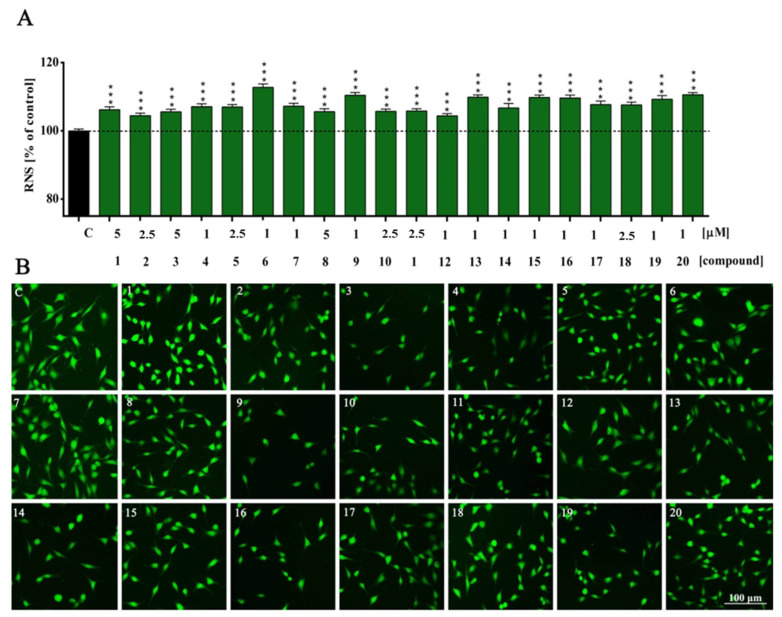
Nitric oxide (NO) level of HT-22 cells treated with compounds **1**–**20** (**A**) for 48 h and representative images (**B**). All experimental groups were compared using one-way ANOVA of variance with Dunnett’s post hoc test; mean ± SD, *n* = 9. Statistically significant results are displayed as: *** *p* < 0.001. Green fluorescence—FITC. Magnification of the objective lens 10×.

In our research, we noted compound-induced accumulation of intracellular nitric oxide (NO). In detail, we observed a statistically significant increase in NO production in all experimental sets (***, *p* < 0.001—compounds **1**, **3**–**11** and **13**–**20**; ***, *p* = 0.0005—compound **2**; ***, *p* = 0.0006—compound **12**). The highest increase in nitric oxide compared to the control group occurs within the derivatives with bigger substituents at both positions, namely, 3-methoxy and 4-methoxybenzyl, and ethoxy and isopropoxy groups (Figure 4, compounds **15**–**16** and **19**–**20**). In contrast, the compounds with furanylmethyl fragments (Figure 4, compounds **1**–**4**) generally were characterized by the lowest impact on the elevation of NO; however, this was still at the significance level. In the case of thiophenylmethyl derivatives, the highest NO was observed in the compound with a methoxy group (Figure 4, compound **6**), while in benzyl derivatives, with no substituted indole (Figure 4, compound **9**).

The NO overproduction we observed is probably directly related to low Ca^2+^ levels. RNS overproduction has been linked with functionally active neurons, increased metabolic activity and cellular plasticity, including neuronal differentiation and neurite outgrowth. Moreover, high RNS levels can influence calcium release and activate signaling cascades directly related to synaptic plasticity. There is, however, a critical limit to the ROS/RNS pool in a cell, which triggers cell senescence and apoptosis. Overproduction can also result in dramatic and long-term changes in cellular excitability and neuronal activity via the calcium-dependent pathway [21,22].

**Figure 5:**
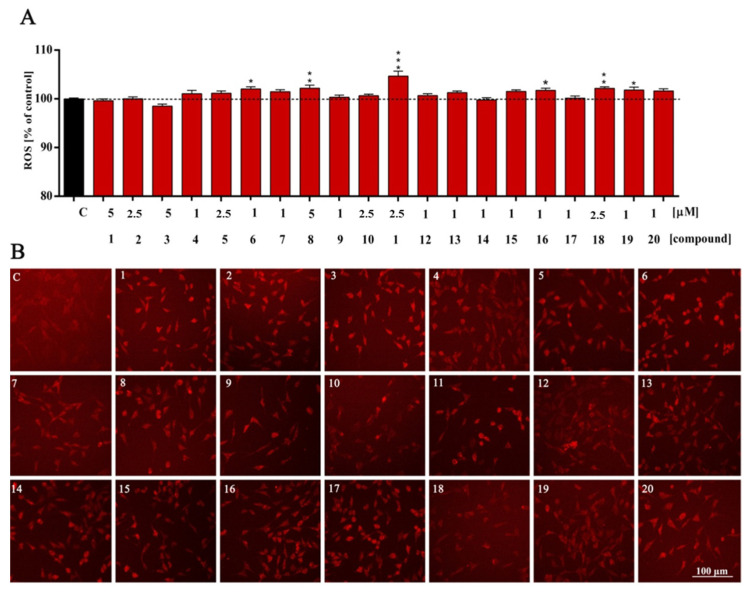
Reactive oxygen species (ROS) level of HT-22 cells treated with compounds **1**–**20** (**A**) for 48 h and representative images (**B**). All experimental groups were compared using one-way ANOVA of variance with Dunnett’s post hoc test; mean ± SD, *n* = 9. Statistically significant results are displayed as: * *p* < 0.05; ** *p* < 0.01; *** *p* < 0.001. Red fluorescence—Texas Red. Magnification of the objective lens 10×.

In addition to RNS, ROS are also necessary for the regulation of physiological cell functions through redox signaling. Here, we observed that compounds tested slightly affect the levels of ROS linked with redox homeostasis. In detail, we noted a significant increase in ROS overproduction only in selected experimental sets. Furanylmethyl derivatives (Figure 5, compounds **1**–**4**) seem to be the most favorable when it comes to affecting the level of reactive oxygen species, causing a slight decrease or no changes (ns, *p* > 0.05). Within them, only the compound with an isopropoxy group at the C5 position of the indole (Figure 5, compound **4**) showed a minor increase in ROS level; however, the result was not statistically significant (ns, *p* > 0.05). The highest statistically significant increase in the level of ROS was observed in the benzyl compound with an ethoxy group at the indole moiety (Figure 5, ***, *p* < 0.0001—compound **11**). The general tendency among all compounds is that a lack of substituent or installation of a small group at the C5 indole (Figure 5, compounds **1**, **2**, **5**, **6**, **9**, **10**, **13**, **14**, **17**, **18**) is more beneficial than the presence of a bigger alkoxy fragment (Figure 5, compounds **3**, **4**, **7**, **8**, **11**, **12**, **15**, **16**, **19** and **20**).

In our study, the ROS level reflected values close to the control (with some exceptions). This may indicate that, in this case, ROS play secondary messenger functions in cell signaling and are essential for various physiological processes. Our research also confirms the general non-cytotoxicity of the tested compounds depending on the structure. ROS levels are often altered by the action of various substances. Recently, ROS have been mainly considered as undesirable oxidative stress-related effects. It turns out, however, that they play an important role as transmitters in redox signaling under normal physiological conditions. Research confirms that ROS accumulation causes damage to various cellular components [23].

**Figure 6:**
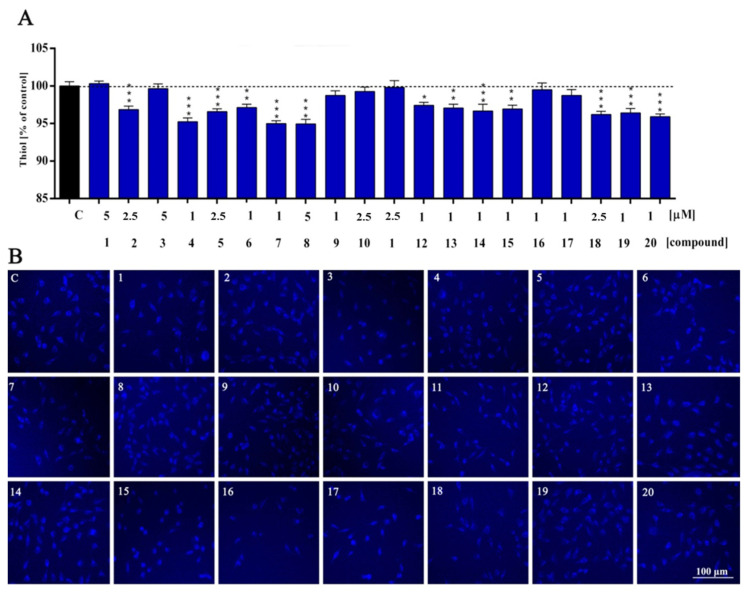
Reduced glutathione (thiol) level of HT-22 cells treated with compounds **1**–**20** (**A**) for 48 h and representative images (**B**). All experimental groups were compared using one-way ANOVA of variance with Dunnett’s post hoc test; mean ± SD, *n* = 9. Statistically significant results are displayed as: * *p* < 0.05; ** *p* < 0.01; *** *p* < 0.001. Blue fluorescence—DAPI. Magnification of the objective lens 10×.

ROS/RNS levels are controlled by intracellular enzymatic and non-enzymatic mechanisms. Here, we observed that compounds tested maintain cellular redox homeostasis by glutathione activation. The cytosolic redox state was possibly compensated by intracellular GSH radical scavenging activity [24]. Considering substituents at the tetrahydropyridine moiety, the biggest decrease in the level of glutathione was caused by compounds possessing the thiophenylmethyl group (Figure 6, ***, *p* = 0.0001—compound **5**; **, *p* = 0.0027—compound **6**; ***, *p* < 0.0001—compounds **7**, **8**), while the lowest decrease was found for derivatives with a benzyl group (Figure 6, compounds **9**–**12**). Installing 3- or 4-methoxy fragments to the benzyl group (Figure 6, compounds **13**–**20**) generally leads to the drop of glutathione level. In the case of substitution at the C5 position of the indole, the biggest impact on lowering GSH level was noticed predominantly in isopropoxy compounds (Figure 6, compounds **4**, **8**, **12**, **16** and **20**). However, interestingly, within 4-methoxyphenyl derivatives, the compound with an isopropoxy group (Figure 6, compound **16**) showed the lowest decrease in GSH.

It is quite clear that GSH function is not limited to defense against free radicals. One of the most important is the involvement of key transcription factors in redox signaling and the detoxification of many exogenous compounds [25].

In conclusion, the depletion of the GSH pool could possibly be due to involvement in RNS/ROS detoxification [26]. However, the mechanism by which RNS/ROS initiate cell signaling may differ. The evidence presented here and in the literature shows that cellular levels of free radicals are strongly associated with the regulation of antioxidant levels in cells. Moreover, previous studies have shown that compounds **5**, **9** and **17** are dopamine D_2_ receptor antagonists [10]. Wei Y et al. demonstrated that the astrocyte dopaminergic receptor D_2_ regulates GSH synthesis [27]. The presented evidence shows that D_2_ receptor activity may also influence the regulation of GSH levels. 

## 3. Materials and Methods

### 3.1. Hippocampal Neuronal Cell Line

Mouse hippocampal cell line (HT-22) (Cat# SCC129, RRID: CVCL_0321, Merck; Kenilworth, NJ, USA) was maintained according to the manufacturer’s recommended medium DMEM (Corning, NY, USA), supplemented with 10% fetal bovine serum (FBS, Gibco; Carlsbad, CA, USA) and antibiotic mix solution (100 U/mL penicillin, 0.1 mg/mL streptomycin) (Thermo Fisher Scientific; Waltham, MA, USA). Cells were grown under standard conditions (37 °C with 5% CO_2_ flow and 95% air humidity (New Brunswick Galaxy 170 R, Thermo Fisher Scientific; Waltham, MA, USA). For assays, cells were seeded at a constant density of 3.0 × 10^3^ cells/cm^2^.

### 3.2. D2AAK1 Derivatives

The synthesis and details of the D2AAK1 derivatives are provided elsewhere [10]. For assays, compounds **1**–**20** were dissolved in DMSO to a stock solution (10 mM). Before each experiment individual dilutions were prepared in a complete culture medium. For each sample tested, the final DMSO concentration (0.5%) was adjusted, which did not affect the viability of the cells. HT-22 cells were treated with different compounds at selected concentrations and incubated for 48 h.

### 3.3. MTT Assay

The assay was performed in accordance with our previous research [8]. Cells were seeded on 96-well plates at standard density. After 24 h, cells were treated with selected concentrations (1–50 µM) of D2 AAK1 derivatives for 48 h. MTT (3-(4,5-Dimethylthiazol-2-yl)-2,5-diphenyltetrazolium bromide) was then added at a final concentration of 0.5 mg/mL. After 4 h of incubation at 37 °C, the absorbance was measured at 590 nm (and 620 nm as a reference) using the BioTek-Synergy H1 microplate reader (Agilent; Santa Clara, CA, USA). Based on the MTT assay, concentrations were selected for further studies.

### 3.4. Cellular Redox Status and Antioxidant Capacity Assessment

The assay was performed in accordance with our previous research [8]. In detail, cells were seeded at standard density on black 96-well fluorescent plates. Measurements were assessed using the following fluorogenic probes at a final concentration of 5 µM: dihydroethidium (Thermo Scientific; Waltham, MA, USA), DAF-2 diacetate (Cayman Chemical; Ann Arbor, MI, USA) and Thiol Tracker Violet (Thermo Scientific; Waltham, MA, USA). Analyses were performed using digital images and quantifications were taken with an InCell Analyzer 2000 (GE Healthcare; Chicago, IL, USA) and presented as relative fluorescence units (RFU).

### 3.5. Intracellular Calcium Analysis

Cells were seeded at standard density and incubated with compounds at selected concentrations for 48 h. Measurements were made using the Fura-2 AM probe (Cayman Chemical; Ann Arbor, MI, USA). Signal detection was performed with an InCell Analyzer 2000 (GE Healthcare; Chicago, IL, USA).

### 3.6. Statistical Analysis

Statistical analysis was performed with GraphPad Prism v. 8.0. All experiments were carried out in triplicate. Differences between the control and study groups were assessed using one-way ANOVA of variance with Dunnett’s post hoc test. All results are presented as mean ± standard deviation. Statistically significant results (*p*-value of <0.05) are displayed as: * *p* < 0.05; ** *p* < 0.01; and *** *p* < 0.001. ImageJ software was used for image processing, while Adobe Photoshop CC software was used to create figures.

## 4. Conclusions

### D2AAK1 versus Its Analogs

Although some D2AAK1 derivatives exhibit a significant increase in metabolic activity, the proliferation rate elicited by the lead structure [8] exceeds that of its most active analog. The level of intracellular calcium is statistically significantly lower after treatment with D2AAK1 [9]. Furthermore, all reported analogs lead to an increase in reactive nitrogen species, while the lead structure causes a drop of this parameter [9]. However, considering the impact on reactive oxygen species and the level of glutathione, there are no significant differences between D2AAK1 and its derivatives (Table 1). In summary, analyzing the structure–activity relationship, we selected the compound with the most favorable profile for neuroprotective activity. Among all implemented structural features, D2AAK1 is the most favorable for neuroprotective activity and recognized as a promising medicinal compound for the treatment of neurodegenerative diseases. Further optimization of D2AAK1 is needed to obtain more potent compounds.

## Figures and Tables

**Table 1:** Summary of statistical differences of all experimental sets. Statistically significant results are displayed as: * *p* < 0.05; ** *p* < 0.01; *** *p* < 0.001.

HT-22																					
	D2AAK1	1	2	3	4	5	6	7	8	9	10	11	12	13	14	15	16	17	18	19	20
**MTT**	***	**	***	***	***	***	***	*	ns	***	***	***	***	*	***	***	ns	***	ns	ns	ns
**Ca^2+^**	***	ns	ns	ns	ns	ns	ns	ns	ns	ns	ns	ns	ns	ns	ns	ns	*	ns	ns	ns	*
**NO**	ns	***	***	***	***	***	***	***	***	***	***	***	***	***	***	***	***	***	***	***	***
**ROS**	ns	ns	ns	ns	ns	ns	*	ns	**	ns	ns	***	ns	ns	ns	ns	*	ns	**	*	ns
**Thiol**	ns	ns	***	ns	***	***	**	***	***	ns	ns	ns	*	**	***	**	ns	ns	***	***	***

## Data Availability

The data will be available on request from the corresponding author.

## Sample Availability

Samples of the compounds of D2 AAK1 derivatives are available from the authors.
